# Moral enhancement and cheapened achievement: Psychedelics, virtual reality and AI

**DOI:** 10.1111/bioe.13374

**Published:** 2024-11-20

**Authors:** Emma C. Gordon, Katherine Cheung, Julian Savulescu, Brian D. Earp

**Affiliations:** ^1^ University of Glasgow Glasgow UK; ^2^ Bloomberg School of Public Health, Berman Insitute of Bioethics Johns Hopkins University Baltimore Maryland USA; ^3^ New York University (NYU) New York New York USA; ^4^ University of Oxford Oxford UK; ^5^ National University of Singapore Singapore

**Keywords:** bioethics, cheapened achievement, human enhancement, moral enhancement

## Abstract

A prominent critique of cognitive or athletic enhancement claims that certain performance‐improving drugs or technologies may ‘cheapen’ resulting achievements. Considerably less attention has been paid to the impact of enhancement on the value of *moral* achievements. Would the use of moral enhancement (bio)technologies, rather than (solely) ‘traditional’ means of moral development like schooling and socialization, cheapen the ‘achievement’ of morally improving oneself? We argue that, to the extent that the ‘cheapened achievement’ objection succeeds in the domains of cognitive or athletic enhancement, it could plausibly also succeed in the domain of moral enhancement—but only regarding certain forms. Specifically, although the value of moral self‐improvement may be diminished by some of the more *speculative* and *impractical* forms of moral enhancement proposed in the literature, this worry has less force when applied to more plausibly viable forms of moral enhancement: forms in which drugs or technologies play an *adjunctive* or *facilitative*, rather than a *determinative*, role in moral improvement. We illustrate this idea with three examples from recent literature: the possible use of psychedelic drugs in certain moral‐learning contexts, ‘Socratic Al’ (a proposed Al‐driven moral enhancer) and empathy enhancement through virtual reality (VR). We argue that if one assumes that these technologies work roughly as advertised (which is an open empirical question), the ‘cheapened achievement’ objection loses much of its bite. The takeaway lesson is that moral enhancement in its most promising and practical forms ultimately evades a leading critique of cognitive and athletic enhancement. We end by reflecting on the potential upshot of our analysis for enhancement debates more widely.

## 1 INTRODUCTION

Biomedical ‘enhancements’ are, on a popular conception, proposed or actual interventions into the psychobiology of a person intended to improve them or their capacities beyond what is needed to treat a disease or pathology.^1^ Such enhancements paradigmatically rely on drugs or technologies developed within biomedical science and can be used to influence many aspects of our lives: examples range from steroid use or other ‘doping’ in elite sport^2^ to ‘study drugs’ to increase focus during exam season^3^ to the use of MDMA or psychedelic drugs (under certain conditions) to try to improve romantic relationships.^4^

Such enhancements are controversial. Take *cognitive* enhancements: interventions to ‘amplify or extend core capacities of the mind through improvement or augmentation’.^5^ According to some authors, so‐called ‘everyday’ (or perceived as ‘normal’)^6^ uses of familiar substances like coffee, traditional educational practices or even browsing the internet^7^ could count as enhancements on this definition, insofar as they boost the mental capacities of healthy individuals. However, philosophical debate has tended to focus on emerging—or established but less familiar—interventions or technologies such as nootropic drugs (especially when used for ‘non‐medical’ purposes), brain–computer interfaces or Artificial Intelligence (AI).^8^ It is, to be clear, attempting to augment human cognition *by such means* that is controversial.

The nature of the controversy depends on the objection. For example, some believe that cognitive enhancement (of such a sort) can amount to a type of *cheating*, at least in certain competitive contexts.^9^ Others fear that it will contribute to the dangerous ‘rat race’ of modern capitalism.^10^ Still others worry that the promotion of cognitive enhancement would exacerbate existing social inequalities (although others claim just the opposite).^11^

However, we will not be concerned with those objections here. Instead, we are interested in a very specific line of critique, which is that cognitive (or indeed, athletic) enhancement ‘cheapens’ associated *achievements*.^12^ Most basically, the worry is that using non‐medically prescribed—or simply not‐perceived‐as‐‘normal’^13^—drugs or technologies to attain a valued aim or objective (such as passing a test) in some way *diminishes the value* of that success, relative to achieving the same success without the enhancer (that is, through the unmediated or less technologically mediated exercise of personal effort and ability). In the domain of sport, this has been dubbed the ‘No pain, no praise’ objection^14^ to bioenhancement.

In Section 2, [Link], we spell out this Cheapened Achievement Argument (CAA) in greater detail. Then, having obtained a better sense of the substantive concerns behind it, we apply it to a topic in the enhancement literature that has so far mostly avoided the CAA treatment: namely, the topic of *moral* enhancement (also termed moral ‘bioenhancement’ or ‘neuroenhancement’).^15^ We ask: Would the use of morally enhancing (bio)technologies, rather than the (exclusive) use of so‐called ‘traditional’ means of moral development such as schooling or socialization, cheapen the ‘achievement’ of becoming a morally better person (or perhaps knock‐on moral achievements enabled by this)? We argue that, plausibly, it would, but only for *certain* types of moral enhancers discussed in the literature: types that we find to be unrealistic. In later sections, we provide examples of what we take to be more realistic types of moral enhancers and explain why the force of the CAA is neutralized or at least substantially weakened in those cases. We conclude by considering whether a similar analysis might apply to other forms of bioenhancement.

But first, we should start with a few words of background on moral enhancement.

### 1.1 Moral enhancement

In the context of debates around cognitive or athletic enhancement, users of certain drugs or technologies are often alleged to be doing something morally questionable: for example, cheating or gaining an unfair advantage over others. In response to this type of claim, Douglas (2008)^16^ introduced the idea of *moral* enhancement to test the limits of bioconservative opposition to human enhancement. In a nutshell, he argued that using a drug or technology to *morally better oneself* can hardly be subject to accusations of cheating (or otherwise harming or disrespecting others) since, on various plausible accounts, to become more moral is precisely to better understand or be able to fulfil one's other‐regarding duties (e.g., by not cheating to get ahead). So, he concluded, if there *were* some principled objection to moral enhancement, it would have to be on some other grounds than that it constitutes or promotes unethical behaviour.

And, indeed, various other grounds have been offered. These have included objections based on meta‐ethical uncertainty or disagreement (e.g., how can we decide if something is a moral enhancer if there is no consensus on what counts as moral improvement in the first place?)^17^; concerns about potentially raising standards for praiseworthiness, leaving the morally unenhanced behind^18^; ignoring structural factors in favour of a focus on individual moral failings^19^; and so on. However, like the objections to cognitive and athletic enhancement mentioned previously, we will not be able to address these alternative objections to moral enhancement here. Instead, our goal is to consider just one objection, the CAA, in relative detail. Accordingly, we will now present a more in‐depth treatment of the argument as applied to various issues (in Section 2, [Link]) before applying it specifically to moral enhancement (Section 3). In Sections 4 and 5, we discuss different *types* of moral enhancement, suggesting that only some fall prey to (the strongest form of) the CAA. In Section 6, we present conclusions.

## 2 THE CAA

The CAA has been advanced against various forms of enhancement in different ways by different authors. Notable examples include Kass (2002), Sandel (2007), Agar (2010) and Harris (2011).^20^ In ‘template’ form, however, the argument goes something like this:

Template CAA

P1) When certain (e.g., athletic or intellectual) aims are accomplished via the use of an enhancer, their being achieved via the substantial exercise of (one's own) effort and ability is thereby undermined.

P2) The value or praiseworthiness of an achievement depends on (requires) the substantial exercise of (one's own) effort and ability.

C) Therefore, when certain aims are accomplished via the use of an enhancer, the value or praiseworthiness of their achievement is undermined.^21^

The argument seems valid as far as it goes; the question, of course, is whether it is sound, and this depends on the strength of each premise. Rationales for P2 can be found within the literature on the value of achievements generally in philosophy. Take the achievement of climbing a mountain as an example. As Bradford (2013)^22^ and Greco (2010)^23^ suggest, actually *climbing* the mountain (rather than just taking a helicopter to the summit) may be of special value insofar as the effortful deployment of one's abilities to reach a desirable end point—above and beyond the sheer fact of reaching the end point—is worthy in its own right. (Perhaps, with Aristotle,^24^ we believe that hard‐earned achievements are an important part of human flourishing.)^25^

However, it is arguments in favour of P1 that are more pertinent to specific debates about enhancement in bioethics. Within the context of these debates, bioconservatives who defend versions of the CAA offer subtly different lines of thinking. Kass,^26^ for example, focuses on how enhancements might disconnect our performances from our efforts, giving us ‘easy’ achievements that are in his view not worth having. In a similar vein, Sandel^27^ argues that cognitive enhancements interfere with the causal link between success and agency, where this link is proposed to be fundamental to the value (and creditability) of our achievements. According to Sandel, famously, if credit is due, it is owed not to the enhanced agent, but ‘to the pharmacist.’

Harris^28^ and Agar^29^ have (in slightly different ways^30^) advanced a modified framing of the problem, whereby the extent to which we value our achievements is bound up with the possibility that we might *fail* to attain them. The risk of failure, in turn, is alleged to be downgraded (or might even be removed altogether) in certain cases of enhancement. Thus, to the extent that an enhancement *does* undermine human fallibility (e.g., by guaranteeing success), it weakens our positive appraisal of the resulting achievement.^31^

Whatever one thinks of the force of these arguments, it is striking that while the CAA has been applied extensively to debates around cognitive or athletic enhancement (e.g., in relation to doping in sport),^32^ it has not yet received much attention in relation to debates around *moral* enhancement.^33^

Why might that be so?

One possibility is that people may find it intuitively odd to speak of ‘achievements’ in the domain of morality, since being or becoming more moral is not traditionally thought of as an ‘accomplishment’ akin to winning a race. After all, the thinking goes, we should try to improve ourselves morally for its own sake or for the sake of others—not because we hope to be congratulated for our efforts or given some sort of prize.

Still, it could be argued that individual moral improvement is something *valuable* and even potentially creditworthy. For example, we might think that a person who goes out of their way to become more moral (*especially* for its own sake or for the sake of others) is deserving of admiration or praise. In that case, it could still make sense to ask whether the *value* or *praiseworthiness* of becoming more moral would be diminished by doing so with the help of a drug or technology as opposed to doing so by sheer dint of will.

Accordingly, whether or not one thinks the CAA cuts ice in relation to cognitive or athletic enhancement, it would still be an open question whether (a transposed version of) the CAA can be successfully applied to moral enhancement. In what follows, then, we explore this question by first clarifying some of the details of the argument, sorting out what a recast version looks like in the case of moral enhancement and determining whether (analogues) of P1 and P2 are plausible once the argument is transposed.

In doing so, we hope to establish two main points: first, that *if* moral enhancement is described in certain fantastical ways (e.g., ‘imagine a pill’‐type scenarios, where the effects of the pill are thought to directly or almost infallibly morally enhance the person), *then* a version of the CAA is probably applicable. However, as we will go on to suggest in our second point, such direct—or externally causally determined—moral enhancement would itself be problematic, both practically and ethically, whereas more realistic and in our view ethically justifiable forms of moral enhancement would be less vulnerable to the CAA.

To illustrate these points, we then outline three examples of what we take to be more viable forms of moral enhancement: that is, forms that do not purport to directly or infallibly morally enhance an individual (for example, by immediately—in either sense of the word—causing them to have morally superior motives or dispositions *however* those might be defined), but that would rather work by other means. For example, they might work by helping individuals to better exercise their agency in recognizing and responding to moral reasons. Also, it is these latter factors that would, or should (if the CAA is to be properly defanged in such cases), remain the primary means by which moral improvement takes place.

Simply put, if a moral enhancer either substantially preserves, or even increases, the ability of the individual to morally improve themselves via means that are normally seen as grounding the value or praiseworthiness of such improvement, then a key assumption of the CAA will not be met.

What would this look like in practice? We propose that any CAA‐proof moral enhancement technology would need to play an *adjunctive* or *facilitative* role in bringing about the changes considered constitutive of moral improvement, thereby preserving (or possibly even amplifying) key aspects of ‘traditional’ moral development such as critical reflection or engagement with reasons: that is, aspects that plausibly *are* a significant part of what makes individual moral improvement widely regarded as something valuable or praiseworthy.

The cases that we consider involve psychedelics, AI and VR. We suggest that, suitably conceived and implemented, none of these forms of adjunctive enhancement would be intrinsically problematic by the lights of a (transposed version of the) CAA. This is because—as we will argue—even if P2 is simply granted in relation to the technologies that we consider, P1 in the transposed version of the argument comes out false.

## 3 APPLYING THE CAA TO MORAL ENHANCEMENT

Let us begin with a thought experiment.

Suppose we have two individuals, Alison and Beth, each of whom wants to improve herself morally. Each has good evidence from friends, family and therapists that the lives they have been leading are suboptimal from a moral perspective. Both are quick to lose their temper, hold petty grudges, give in to knee‐jerk xenophobic tendencies, cheat on their taxes and partners and so on.

Each is offered a special ‘pill’ that, if taken, instantly causes the user to be slow to anger, quick to forgive, tolerant, inclusive, honest and loyal. Suppose Alison, declining the pill, spends years working on her character, practicing altruism and reading and absorbing moral wisdom from different cultures. Through dogged commitment, sustained reflection and effort in practice, Alison slowly begins to habituate more prosocial patterns of behaviour, to internalize relevant moral norms and to be moved in a way that she was not previously by moral reasons (reasons that she is now more sensitive to as well). Through all this dedication and effort, suppose Alison, by the end of the story, has undergone a successful journey of moral self‐improvement.

Beth, by contrast, just cannot be bothered with all that hard work and simply takes the pill, which remarkably (never mind how) directly causes her to end up with behavioural dispositions, attitudes, moral beliefs and so on exactly similar to Alisons's.

What should we say about these two contrasting cases?

The answer depends, in part, on whether we accept the stated stipulations. However, given the fantastical nature of Beth's case, we might struggle or refuse to do so. Look, someone might say, I do not care how it supposedly ‘works’—any mere pill that quickly and effortlessly converts you into a supposedly more moral being just would not (regardless of what you armchair philosophers are asking me to imagine) bring about as *reliable* or *enduring* or *deeply rooted* a moral change as what years of hard work and reflection would do. So, this objection continues, the two cases are not relevantly similar: they would not have a similar outcome. We should therefore reject moral bioenhancement, since any such ‘quick‐fix’ approach to the issue would fail to result in moral improvement of a similar quality or stability to what can be brought about by traditional means. After all, what is quickly or effortlessly gained may be just as quickly or effortlessly lost.

Perhaps that is right. But see if you can get yourself to accept the empirical stipulation. We are supposing here that by popping a pill, Beth *really would* end up in an identical state to Alison in terms of moral beliefs, attitudes, dispositions, behaviour and so on, *including* in terms of reliability, durability, quality, stability, deep‐rootedness and all the rest. In that case, would there still be a reason to reject ‘the pill’?

From a bioconservative perspective, it seems there might be. Specifically, it might be thought that Beth's achievement of moral improvement is somehow *cheapened* compared to Allison's, despite the fact that (*ex hypothesi*) both of their moral starting points and moral end points are the same. Someone who is moved by this response might be tempted to reason along the following lines, thereby embracing a moral enhancement version of the CAA:

Template CAA (Moral Enhancement Version)

P1‐ME) When certain moral aims (e.g., moral self‐improvement) are accomplished via the use of an enhancer, their being achieved via the substantial exercise of (one's own) effort and ability is thereby undermined.

P2‐ME) The value or praiseworthiness of a moral achievement depends on (requires) the substantial exercise of (one's own) effort and ability.^34^

C‐ME) Therefore, when certain moral aims are accomplished via the use of an enhancer, the value or praiseworthiness of the moral achievement is undermined.

Given the prima facie plausibility of P2‐ME (and some preliminary empirical work suggesting that it might be commonly endorsed by laypeople, at least in Germany),^35^ what shall we say about P1‐ME? Based on our thought experiment involving Allison and Beth, especially Beth's case, it might certainly seem that we have a reason to endorse P1‐ME, and so, the conclusion C‐ME.^36^

However, the situation is not that straightforward. We need not accept the conclusion C‐ME (see Box 1 for how we might respond if we *did* accept C‐ME). Our reasoning here has two main steps. First, we explain why the ‘magic pill’‐type thought experiment that we used—namely, one that might seem to motivate P1‐ME—is actually quite misleading. It relies on a mistaken idea that moral enhancement, even of a relatively high‐tech or nontraditional kind, would best be understood as playing a ‘determinative’ role in enhancing us morally, for example, that moral enhancements ‘will somehow cause moral improvement all on their own.’^37^ As we will see in the following sections, this assumption is mistaken. To be either realistic or desirable, we suggest that moral enhancement should involve the *adjunctive* use of a drug or technology to complement and/or render more effective so‐called ‘traditional’ modes of moral improvement: that is, modes that retain putatively valuable features, such as reflective engagement with moral reasons.

Box 1. **Clarifying the aim of the argument: *Ceteris paribus* or all‐things‐considered?**

In the main text, we explain why we do not accept the conclusion C‐ME. However, it is important to clarify the wider landscape of this debate. For, even if we *did* accept C‐ME, this would not (all by itself) entail that moral enhancement was impermissible. For example, it might turn out that, even if the use of moral enhancers *did* cheapen the achievement of morally bettering oneself, it should still be done *anyway*.^38^ For instance, one might think (e.g., along the lines of Persson and Savulescu)^39^ that the beneficial consequences of moral enhancement (suitably pursued) could *justify* the cheapening of whatever associated achievement on an individual level. As the thought might go: the particular success of moral improvement is so important to attain that it matters less *how* we attain it than *that* we attain it. Consider: How many people should *die* or *suffer mistreatment* while Alison takes time to effortfully improve herself? Plausibly, the answer is ‘None.’^40^ According to this way of seeing things, given the stakes of moral enhancement, it might still be justifiable even if it did diminish the level of praise owed to each individual.

However, we must be careful not to interpret proponents of the CAA as necessarily denying this. In other words, we do not claim that proponents of the CAA would necessarily adopt the further premise that *all‐things‐considered*, we should not pursue a given moral aim through enhancement whenever use of that enhancement would cheapen the attainment of that moral aim. A more charitable interpretation of the argument, we think, is that accepting the conclusion C‐ME would give one at least a *ceteris paribus* reason to refrain from moral enhancement. That is, if C‐ME is true, and moral enhancement cheapens moral achievement, then *all else equal*, we should refrain from moral enhancement. In short, we should construe the argument as at most offering a *pro tanto* (defeasible) rather than *ultima facie* reason to refrain from moral enhancement.

However, once we see how ability and effort would, and should, realistically be involved in the ‘adjunctive’ use of a moral enhancer, we find that we have good reason to reject P1‐ME (and so, the conclusion C‐ME). We conclude that there may be no special value to moral improvement *absent*, rather than *accompanied by*, ‘high‐tech’ facilitators of moral growth, insofar as these facilitators play an *adjunctive* role in moral enhancement as we will describe.

## 4 AGAINST DETERMINATIVE MORAL ENHANCEMENT

Some who object to the idea of moral (bio)enhancement seem to do so, at least in part, because they envision that such enhancement would work by *directly*, *comprehensively* and/or *certainly* bringing about particular desired moral changes in the agent (e.g., a tendency to be less hot‐tempered) without a need for the agent to actually grasp the moral reasons that they have for altering their behaviour, similar to the God Machine proposed by Savulescu and Persson.^41^ As Jotterand (2011)^42^ argues, ‘[w]hile the manipulation of moral emotions might change the behaviour of an individual, it does not provide any content, for example, norms or values to guide one's behavioural response’ (6, see also 8). Or as Sparrow (2014)^43^ suggests: ‘It is hard to see how any drug could alter our beliefs in such a way as to *track the reasons* we have to act morally’ and that ‘someone who reads Tolstoy arguably learns reasons to be less judgmental and in doing so develops greater understanding: someone who takes a pill has merely caused their sentiments to alter.’^44^

But, as one of us has put it elsewhere, ‘what about reading Tolstoy *while* taking a pill (i.e., a pill that enhances one's moral learning vis‐à‐vis the text)?’^45^ (emphasis added). The supposition here is that this hypothetical pill would ‘occasion a state of mind that made the moral lessons of Tolstoy more apparent or more compelling to the reader.’^46^ Thus, a robust educational or learning context would still be needed. What is proposed is a *facilitating* rather than *determining* role for any high‐tech enhancer,^47^ such that it would preserve a role for critical engagement with reasons^48^ as well as some kind of actual moral ‘content’^49^ (e.g., ‘norms or values’).

However, things may not be quite so simple. Consider this objection^50^ to our proposed distinction between ‘direct’ or ‘determinative’ moral enhancement (which might *well* cheapen the associated moral achievement) and adjunctive–facilitative moral enhancement (which we are suggesting would evade the thrust of the CAA):

if someone is quick to anger, say, then it is likely that this will get in the way of their ability to engage with moral reasoning, at least in the cases where their temper has kicked in. So then, it would be reasonable to assume that an intervention that would allow them to be less quick to anger—even a seemingly ‘direct’ or ‘determinative’ one—would enable them to take a step back and engage with moral reasons before acting. But then why should this not be considered adjunctive–facilitative rather than direct or determinative? After all, they may still make the same moral decision but now guided by their own moral reasoning rather than being propelled by emotion and a knee‐jerk angry reaction.

In line with this objection, suppose someone *is* directly caused to be less quick‐tempered (i.e., by some deterministic intervention), rather than by an indirect, adjunctive–facilitative intervention that works by, say, enabling them to grasp or appreciate *why* being quick‐tempered is a problem. In this case, it might well be that the cheapened achievement objection applies, but only narrowly: that is, *having become less quick‐tempered* is not something for which the individual can take personal credit (apart, perhaps, from having decided to do something drastic to address their anger problem in the first place, namely, by choosing to undergo the sure‐fire intervention—a decision that might be praiseworthy in its own right compared to certain alternatives such as doing nothing, or, say, repeatedly trying but failing to put a reign on their temper). Nevertheless, *once they have been deterministically induced to be less quick‐tempered*, in this imagined example, it could be the case that, as a result of this, they are in a better position to reflect on moral reasons (i.e., more generally) and so morally enhance themselves in a way that is creditworthy, albeit along *other* dimensions, or in *other* respects.

Or let us use a real‐life example. Suppose a person diagnosed with ADHD, who is prone to violent outbursts, takes methylphenidate (Ritalin) and finds that they are, by virtue of taking the pill, instinctively less prone to reacting angrily such that they can better consider the long‐term consequences of their actions.^51^ If so, it might well be the case that, in respect of that particular change (i.e., being less prone to anger), their achievement is cheapened; however, this would still leave plenty of room for more global improvements that need not be cheapened in the same way. In the following sections, we discuss further examples of more realistic moral enhancers.

## 5 ADJUNCTIVE–FACILITATIVE MORAL ENHANCEMENT

Here, we outline three potentially promising forms of adjunctive–facilitative moral enhancement: psychedelics, Socratic AI and VR. All three methods, as we are describing them, play a facilitating, rather than a determinative role in moral enhancement and therefore leave space for critical engagement with reasons. It is important to note that the applications that we discuss here are all instances of voluntary moral self‐enhancement: for now, we are setting aside any discussion of involuntary moral enhancement (e.g., for violent psychopaths).^52^

### 5.1 Psychedelics

There is now a burgeoning literature on the prospect of using certain psychedelic (or similar) drugs *in conjunction* with other practices, such as psychotherapy or community‐based healing or spiritual observances, to bring about altered states of consciousness and emotion. The drugs in question include psilocybin (from “magic” mushrooms), lysergic acid diethylamide (LSD) or 3,4‐Methylenedioxymethamphetamine (MDMA, commonly known as “Ecstasy“ or “Molly”). The altered states that these drugs can bring about are alleged to be capable of helping a person ‘see the world differently’ from how they would during normal waking consciousness, allowing them, in some cases, to better grasp or internalize what many would regard as genuine moral insights.^53^

An intriguing example comes from a study by Noorani et al. (2018), who interviewed participants in a psilocybin‐assisted smoking‐cessation therapy trial about their subjective experiences during, and thoughts and reflections following, the trial: ‘Participants reported gaining vivid *insights* into self‐identity and *reasons* for smoking from their psilocybin sessions. Experiences of interconnectedness, awe, and curiosity persisted beyond the duration of acute drug effects’^54^ (emphasis added). Note the explicit mention of gaining self‐insights and reasons (not simply behavioural changes, for example, automatically stopping smoking), along with a shift in mindset towards awe and curiosity that enabled some participants to view old habits and patterns in a new light and also to *reflect* on what they saw from this angle. One participant reported coming to a profound realization during her second psilocybin session that smoking did not have to define who she was:

For a few seconds, it was just like ‘I'm me, and there are no defining characteristics!’… that made me realise that I'm not a ‘smoker’.^55^

Emotional shifts, including towards a feeling of greater interconnectedness, seemed to facilitate insights—that is, a grasping of *reasons* to change one's behaviour, along with an increased motivation to do so—for several participants. For example, one of them explained:

I had always had the sense of everything being connected. And [the psilocybin session] reinforced that, very strongly… [If I were to smoke] I would be a polluter…ashtrays and butts all over the place, and you're causing harm to other people's health as well. And so you were re‐looking at your place in the universe and what you were doing to help or hinder it. The universe as such. And by smoking, you wouldn't be helping.^56^

These are, of course, selected quotes from a single study: i.e., anecdotes. However, arguments and evidence are accumulating, more broadly, that in supportive settings and with careful preparation, psychedelics have the *potential* to work as facilitative–adjunctive moral enhancers for some individuals, possibly by increasing the ability to adaptively modulate one's moral and emotional responses across a range of settings.^57^ In an influential double‐blind clinical study, psilocybin ‘occasioned experiences […] which were rated by volunteers as having substantial personal meaning and spiritual significance’ and led to sustained positive changes in attitudes and behaviours, including increased patience, playfulness, mental flexibility, optimism, interpersonal perceptiveness and care and compassion or social concern.^58^ While some of these changes might seem to constitute moral improvements in and of themselves (e.g., being more caring or compassionate), others (e.g., patience, playfulness or mental flexibility) might rather seem more like *tools* or *capacities* that one could use in a longer term project of moral self‐development: one that would require deliberate effort, reflection, practice and emotional engagement—not simply passive acquiescence.

One proposal suggests that moral enhancement should target (excessive) self‐interest in order to promote prosocial behaviour or altruism.^59^ On this view, increasing the motivation of some agents to benefit others may (depending on the agent's starting disposition, socio‐relational circumstances and so on) count as a form of moral improvement,^60^ at least according to various plausible views about the relationship between cultivating a less egocentric, more prosocial orientation and the development of a good moral character. Psychedelics, if taken under the right conditions, may be able to assist with such an aim for some people, insofar as they can cause a diminished sense of self at high doses, thus reducing ‘egocentric attributions of salience and enhance[ing] non‐egocentric attention to the world.’^61^

However, again, simply *having* such an experience does not entail that one is now automatically morally enhanced. Rather, having experienced what some describe as ‘self‐transcendence’ under the influence of the drug, the individual may, upon returning to an ordinary state of consciousness, *recall* what the experience was like (e.g., how it felt to intuitively place greater weight on the welfare interests of others), and then *practice* seeing things from that perspective (and acting accordingly) going forward. If one does not take those active steps, however, it may be less likely that any real moral improvement would occur or at least be sustained.

Similarly, psychedelics may foster significant feelings of unity and connectedness, including, in some users, a sense of nature‐connectedness, which has been hypothesized to promote pro‐environmental attitudes and behaviours (although existing evidence is primarily correlational, derived from open‐label pilot studies with small sample sizes, or otherwise preliminary and limited).^62^ Moreover, the acute subjective experience of psychedelics—which includes the altered states of perception—may play a role in helping the agent to actively engage with, make sense of and effectuate certain desired changes (e.g., in relation to therapeutic aims).^63^

Finally, psychedelics may also help some people remember or better appreciate their *existing* values, from which they may have become disconnected. For example, one participant in the aforementioned smoking‐cessation trial said ‘I don't know if I really learned—it was more like letting back in stuff that I had blocked out?… I don't think I changed my values, just remembered more of them. Or just remembered to honour them more, or… allow them more.’^64^ Such qualitative data highlight the adjunctive role that psychedelics may play in moral enhancement for some people: instead of entirely new or alien values being ‘directly’ implanted into the agent, psychedelics may instead promote the ability of some agents to centre moral values that they already possessed or to increase the salience of moral goals that they were already pursuing.

It is important to underscore that psychedelics do not *inevitably* lead to moral improvement (again, however that is defined), nor to the development of certain characteristics. Thus, although popular media articles and some psychedelic enthusiasts have contributed to ‘hype’ around psychedelics,^65^ insisting that they can improve, or even radically transform, society, such apparent cheerleading must be treated with scepticism. Seeking to counteract such biased enthusiasm, Pace and Devenot (2021),^66^ for instance, have highlighted stories of psychedelics users who remained authoritarian in their views post‐psychedelic use or who became radicalized after their psychedelic experience. In line with this, they suggest that psychedelics may act as *non‐specific amplifiers*: that is, they can give rise to shifts in various directions, depending on the individual's personality, their mindset or attitude going into the experience, and the social, political, ideological or institutional context in which the drugs are used.

However, this non‐determinative nature precisely supports our point: it underlines the *facilitating* role that psychedelics may one day play in moral enhancement, rather than a determinative one: individuals neither immediately nor inevitably adopt certain characteristics, attitudes or behaviours post‐psychedelic use. Rather, their effects may depend to a large extent on what the individual brings to the experience (e.g., in terms of motivations, aims, intentions and willingness to productively follow up on any perceived moral insights), the social and relational dynamics of the experience (including any vulnerabilities, such as susceptibility to manipulation) and other important factors, many of which are still only poorly understood.

### 5.2 Empathy training with VR

Turning to a different technological method that does not directly alter the brain, empathy training with VR may one day present an additional avenue for adjunctive moral enhancement: that is, an approach that would preserve room for reflection, effort and critical engagement in the process of seeking moral improvement. The primary medium for ‘entering’ VR involves a head‐mounted display (HMD), commonly referred to as VR glasses or goggles. Complementing this, haptic devices like gloves, bodysuits and haptic‐enabled boots enhance the VR encounter by offering sensory feedback to users.

VR systems offer three key features that some researchers believe could be valuable for empathy training: immersion, presence and embodiment. Immersion refers to the feeling of being completely absorbed in the virtual experience. Users often feel so engaged that they forget about their real surroundings. Presence refers to the convincing illusion that you are physically present in the virtual environment, rather than just viewing it on a screen. And embodiment is the sensation that the virtual avatar (your digital representation in the VR world) is your own body. Users often feel as if they are inhabiting the avatar's form. These characteristics allow users to have deeply engaging and realistic experiences in VR, which could potentially be harnessed to create powerful empathy‐building scenarios.^67^

To clarify how empathy might be enhanced through VR use in a way that would plausibly count towards moral improvement, Zahiu and colleagues distinguish between *bounded* and *reflective* empathy. They write that ‘bounded empathy is the result of how evolutionary forces shaped human moral psychology through time, whilst reflective empathy uses reasoning, moral understanding and emotional regulation when purposefully imagining how it is to be in somebody else's shoes.’^68^ It is the latter form of empathy that VR might be well positioned to enhance, as reflective empathy requires vivid representations of the experiences of others. Here, VR can help to scaffold our imaginative powers, providing a new medium for perspective‐taking by attaching sensory representations to certain experiences that previously had to be mentally conjured. This can be combined with explicit moral reasoning tasks, as shown in a recent study by Dunivan and colleagues (2024). Although they did not find evidence of a ‘direct’ shift in empathy or compassion after viewing a VR‐enhanced film depicting the experience of refugee children (versus a VR‐enhanced control film), they did find significant changes ‘in *moral reasoning* from personal interest to post‐conventional stages [i.e., seeking moral principles beyond those derived from authority and that apply beyond one's own identity group], and a significant increase in the Care/harm factor of moral foundations’^69^ as measured by the Moral Foundations Questionnaire.^70^

Just as with psychedelics (or reading Tolstoy), then, VR might play a *facilitating* role in a wider process of moral enhancement, here, focused on the development of ‘reflective’ empathy, which draws on moral reasoning as well as emotional engagement. In VR, the agent is not compelled to make a choice, not to be more (or unthinkingly) empathetic. Rather, by enhancing our ability to imagine the perspectives of others, while also being prompted to reason about the implications of what we see from those perspectives, appropriately designed and structured VR sessions could allow for both emotional stimulation and critical engagement with the values, beliefs, feelings and attitudes that one might wish to develop.

### 5.3 Socratic AI

The final potential adjunctive–facilitative enhancer that we will briefly canvass is that of AI, specifically in the form of a Socratic Assistant for moral enhancement. AI here refers to ‘a system that collects information from multiple sensors and databases to process it according to its functional relevance for the system user,’^71^ with a growing literature on the potential to use AI, including specially adapted large‐language models,^72^ for moral enhancement.^73^ Particularly relevant to the current discussion on adjunctive moral enhancement is a proposal set out by Lara and Deckers, ‘Socratic Enhancement,’ which aims to address some of the all‐too‐human cognitive limitations that impede moral reasoning and development. Their proposal consists of a Socratic AI Assistant, which would aid the agent in reaching a better decision. Specifically, it would help them learn to reason ethically through constant dialogue between the agent and the machine (previously described as a form of Moral AI).^74^ The machine would not be committed to any pre‐designed ethical perspective: its goal would be to help users develop their own moral values and reasoning, rather than simply informing the agent which action would be ‘ethical’ or not. This dialogue between agent and machine would take the form of the machine asking relevant questions, providing information and revealing failures in argumentation to the agent.

Due to the active role of the agent in this process of development, Socratic AI falls in the category of a potential form of adjunctive moral enhancement (referred to by Lara and Decker as ‘auxiliary enhancement’). The agent deliberates in dialogue with the machine, and is able to accept or reject the advice of the machine before making a decision: as Lara and Deckers write, the agent is intended to have the ‘first and last word’ in the process.^75^ Just as with psychedelics and VR, Socratic AI will not directly or deterministically alter the age morality: instead, the technology only enhances their ability to overcome cognitive limitations in the moral domain and therefore to reach better moral judgements.

### 5.4 Stepping back

Let us now step back and see what we may have learned. The three example cases just canvassed offer a useful perspective for revisiting (P1‐ME), which states that when a moral aim is attained through enhancement, the extent to which it is attained through ability and effort is thereby diminished. Let us now distinguish two distinct claims that (P1‐ME) implies:

(P1‐ME*) When certain moral aims (e.g., moral self‐improvement) are accomplished via the use of a *determinative moral enhancer*, their being achieved via the substantial exercise of (one's own) effort and ability is thereby undermined.

(P1‐ME**) When certain moral aims (e.g., moral self‐improvement) are accomplished via an *adjunctive–facilitative moral enhancer*, their being achieved via the substantial exercise of (one's own) effort and ability is thereby undermined.

Given that moral enhancement can in principle be (at least) either determinative or adjunctive–facilitative, PI‐ME is true only if both (P1‐ME*) *and* (P1‐ME**) are true. However, even if (P1‐ME*) is true (and we have suggested that it is), the examples in this section offer a presumptive case for thinking that (P1‐ME**) is false. Whereas determinative moral enhancement would, *ex hypothesi*, bypass effort, ability, engagement and/or reflection, we have argued that adjunctive–facilitative moral enhancement, as illustrated by the above examples, is best understood as being predicated upon such input and investment from the agent. Put another way, not only does adjunctive–facilitative moral enhancement not undermine effort, ability and so on, as deployed in the service of attempted moral improvement, but rather, such deployment is *required* in such cases for the potential enhancer to facilitate moral improvement at all.

In the psychedelic case, the intended outcome would arise, not through simply taking a drug, but rather through preparing oneself for a drug‐facilitated experience of deliberate moral investigation under appropriate conditions (perhaps something like the supported use model of supervised legal consumption being trialled in the U.S. state of Oregon)^76^ and then seriously reflecting on any apparent moral insights gained, discussing and debating them with others and actively practicing and implementing any warranted changes in attitude, perspective or behaviour.

Likewise in the VR case: simply being connected to the medium itself has no intrinsic ability to foster moral improvement; its usefulness depends entirely on the ability and effort of the agent engaged in the relevant tasks designed to elicit empathy, improve perspective‐taking and practice moral reasoning. A similar lesson applies to the Socratic AI case. One can no more improve oneself morally via Socratic AI without investing the requisite effort or reflection than one can, in a non‐enhanced case, morally improve oneself by failing to engage seriously in the process of dialogue with one's Socratic interlocutor. Any role that such an interlocutor might play in bringing about moral improvement thus *requires*, not *replaces*, the need for such engagement.

In sum: (P1‐ME**) is false,^77^ and so (P1‐ME) is false. The most viable forms of moral enhancement, adjunctive–facilitative moral enhancement, thus escape the conclusion of the cheapened achievement objection even if (P2‐ME) is accepted.

There is, however, at least one relevant objection. It may be that in some cases, a moral enhancer intended to be adjunctive–facilitative could end up enabling an agent to (relatively) effortlessly access, or be persuaded or motivated by, genuine moral reasons. They just ‘see things in another light.’ In *such a case*, would it not be right to say that the individual's moral achievement (or the value or praiseworthiness of the achievement) had been truly undermined?

Elsewhere, Maslen, Savulescu and Hunt (2020) have argued, albeit specifically in relation to enhancement in sport, that it is *not* in fact effort per se that matters for praiseworthiness, but rather costly commitment to a worthwhile goal.^78^ They give the example of Sartre, who reportedly had to decide between fighting for the French Resistance in World War 2 or staying at home to look after his mother. Such a decision might be made instantly and effortlessly. But that does not make it any less praiseworthy if it were made for good or justifiable reasons. The “cost” in this case is the opportunity cost of having to forgo another valuable pursuit.

In the case of moral enhancement, the ‘cost’ will frequently be pursuing one's own self‐interest or one's own autonomously chosen projects. These represent relevant sacrifices and demonstrate sufficient costly commitment. And, in this case, the worthwhile goal is the responsiveness to moral reasons. So, *even if* adjunctive–facilitative moral enhancement comes easily and instantly in some cases, it could *still* potentially be praiseworthy, on a transposed version of Maslen et al.'s account, insofar as it represents a costly commitment to the worthwhile goal of moral improvement. Similar arguments might apply to cognitive enhancement as well.

## 6 CONCLUDING REMARKS

Existing literature on human enhancement in connection with the value of achievement has focused on athletic and cognitive achievement; a prevailing line of argument in this space has been that drugs and technologies that improve performance in physical and cognitive domains may do so at the risk of ‘cheapening’ our resulting (physical and cognitive) achievements.

Given that we have good reasons to want to improve our moral capacities as well, an important but thus far largely overlooked question concerns whether or to what extent bioenhancement would undermine (and if so under what conditions) our *moral achievements*. This paper aimed to put our understanding of this question on a new footing, by first (i) showing that moral achievements are not in principle immune from the cheapened achievement objection, and (ii) this is particularly so in the case of *determinative* moral enhancement. However, determinative moral enhancement is itself not the most promising approach to moral enhancement. More viable by comparison is *adjunctive–facilitative* moral enhancement, which is itself much more resilient to the cheapened achievement objection. We discussed in some detail three plausible or potential forms of such enhancement that illustrate our position—viz., adjunctive use of psychedelic drugs in certain moral‐learning contexts, ‘Socratic AI’ (a proposed AI‐driven moral enhancer) and ‘reflective’ empathy or moral reasoning training through VR—all of which are either currently available or in various stages of development, and all of which are, we have suggested, largely *predicated* upon the exhibition of ability, engagement/reflection and effort. Finally, we have argued that even if such enhancements do reduce effort in certain cases, the agent might still be praiseworthy insofar as they show a costly commitment to the worthwhile goal of moral improvement, including by means of moral enhancement. The takeaway lesson is that moral enhancement in its most promising and practical forms ultimately sidesteps what has been, in the cognitive and athletic enhancement debates, a key line of critical resistance.

## Footnotes

1 For a summary of different definitions of ‘enhancement’ used in bioethics, see, for example, Juengst, E., & Moseley, D., (2019). Human enhancement. In E. N. Zalta (Ed.), *The Stanford encyclopedia of philosophy*, Summer 2019. Metaphysics Research Lab, Stanford University; or Earp, B. D., Sandberg, A., Kahane, G., & Savulescu, J. (2014). When is diminishment a form of enhancement? Rethinking the enhancement debate in biomedical ethics. *Frontiers in Systems Neuroscience*, *8*(12), 1–8. Despite a diversity of definitions, however, the example interventions discussed in this paper should qualify as enhancements on most or all of the major proposals.

2 See, for example, Holm, S., & McNamee, M. (2011). Physical enhancement: What baseline, whose judgment? In J. Savulescu, R. T. Meulen, & G. Kahane (Eds.), *Enhancing human capacities* (1st ed., pp. 291–303). Wiley; Miah, A. (2011). Physical enhancement: The state of the art. In J. Savulescu, R. T. Meulen, & G. Kahane (Eds.), *Enhancing human capacities* (1st ed., pp. 266–273). Wiley; Savulescu, J., Foddy, B., & Clayton, M. (2004). Why we should allow performance enhancing drugs in sport. *British Journal of Sports Medicine*, *38*(6), 666–670.

3 E.g., Cakic, V. (2009). Smart drugs for cognitive enhancement: Ethical and pragmatic considerations in the era of cosmetic neurology. *Journal of Medical Ethics*, *35*(10), 611–615; Mohamed, A. D. (2014). Neuroethical issues in pharmacological cognitive enhancement. *Wiley Interdisciplinary Reviews: Cognitive Science*, *5*(5), 533–549.

4 For recent work, see Earp, B. D., & Savulescu, J. (2020). Psychedelic relationship enhancement. *Philosophy and Public Issues*, *10*(3), 3–28; Earp, B. D., & Savulescu, J. (2020). What is love? Can it be chemically modified? Should it be? *Philosophy and Public Issues*, *19*(2), 93–125; and Buyukbabani, M. B., Earp, B. D., Hannikainen, I., Barba, T., Mihailov, E., Yaden, D., & Savulescu, J. (2024). Moral attitudes toward pharmacologically assisted couples therapy: An experimental bioethics study of real‐world ‘love drugs’. *AJOB Neuroscience*, *15*(4), 239–243. See also Earp, B. D., & Savulescu, J. (2020). *Love drugs: The chemical future of relationships*. Stanford University Press.

5 Bostrom, N., & Sandberg, A. (2009). Cognitive enhancement: Methods, ethics, regulatory challenges. *Science and Engineering Ethics*, *15*, 311–341.

6 ‘Normal’ can be interpreted both descriptively and prescriptively; however, these two senses are often intertwined. See Martín, D., Rueda, J., Earp, B. D., & Hannikainen, I. R. (2023). Normality and the treatment‐enhancement distinction. *Neuroethics*, *16*(13), 1–14.

7 Voinea, C., Vică, C., Mihailov, E., & Savulescu, J. (2020). The internet as cognitive enhancement. *Science and Engineering Ethics*, *26*(4), 2345–2362.

8 This critical resistance might initially sound puzzling, as there are many reasons why the use of cognitive enhancements looks promising and on the whole positive. After all, if we are able to improve our ability to process information, increase our memory and sharpen our focus, we will surely be better positioned to meet personal goals (e.g., enhancing skills that are helpful to our hobbies or careers) as well as goals that matter for wider human flourishing and survival (e.g., developing cures for future pandemics and better addressing the threat of climate change, etc.). Even so, criticism of cognitive enhancement points to various risks or downsides, as noted in the main text. For a discussion of recent developments, see, for example, Saha, S., Mamun, K. A., Ahmed, K., Mostafa, R., Naik, G. R., Darvishi, S., Khandoker, A. H., & Baumert, M. (2021). Progress in brain computer interface: Challenges and opportunities. *Frontiers in Systems Neuroscience*, *15*, 578875; and for a primer on the ethical issues presented by current and likely future nootropic drugs, see for example, Maslen, H., Faulmüller, N., & Savulescu, J. (2014). Pharmacological cognitive enhancement—How neuroscientific research could advance ethical debate. *Frontiers in Systems Neuroscience*, *8*, 107. Meanwhile, see Porsdam Mann, S., Earp, B. D., Møller, N., Vynn, S., & Savulescu, J. (2023). AUTOGEN: A personalized large language model for academic enhancement—Ethics and proof of principle. *The American Journal of Bioethics*, *23*(10), 28–41, and Porsdam Mann, S., Earp, B. D., Møller, N., Suren, V., & Savulescu, J. (2024). AUTOGEN and the ethics of co‐creation with personalized LLMs—Reply to the commentaries. *The American Journal of Bioethics*, *24*(3), 6–14, for work on large language models for academic enhancement.

9 For discussion, see, for example, Porsdam Mann, S., de Lora Deltoro, P., Cochrane, T., & Mitchell, C. (2018). Is the use of modafinil, a pharmacological cognitive enhancer, cheating? *Ethics and Education*, *13*(2), 251–267. See also Schermer, M. (2008). On the argument that enhancement is ‘cheating’. *Journal of Medical Ethics*, *34*(2), 85–88.

10 Sparrow, R. (2015). Enhancement and obsolescence: Avoiding an “enhanced rat race”. *Kennedy Institute of Ethics Journal*, *25*(3), 231–260.

11 See, for example, Ray, K. S. (2016). Not just “study drugs” for the rich: Stimulants as moral tools for creating opportunities for socially disadvantaged students. *The American Journal of Bioethics*, *16*(6), 29–38. Of course, there are other objections as well. For some reasons to worry about cognitive enhancement's impact on authenticity, for example, see, for example, Bublitz, J. C., & Merkel, R. (2009). Autonomy and authenticity of enhanced personality traits. *Bioethics*, *23*(6), 360–374; Juth, N. (2011). Enhancement, autonomy, and authenticity. In J. Savulescu, R. T. Meulen, & G. Kahane (Eds.), *Enhancing human capacities* (1st ed., pp. 34–48). Wiley. Though see also Gordon, E. C. (2022). Trust and psychedelic moral enhancement. *Neuroethics*, *15*(2), 19; Pugh, J. (2020). *Autonomy, rationality, and contemporary bioethics*. Oxford University Press; for (qualified) defenses against the authenticity worry. For a useful overview of the inequality objection and a distribution proposal that aims to avoid inequality, see Giubilini, A., & Minerva, F. (2019). Enhancing equality. *The Journal of Medicine and Philosophy: A Forum for Bioethics and Philosophy of Medicine*, *44*(3), 335–354.

12 For discussion, see Gordon, E. C. (2022). Cognitive enhancement and authenticity: Moving beyond the impasse. *Medicine, Health Care and Philosophy*, *25*(2), 281–288.

13 See Martín, D., et al., op. cit. note 6.

14 Maslen, H., Savulescu, J., & Hunt, C. (2019). Praiseworthiness and motivational enhancement: ‘No pain, no praise’? *Australasian Journal of Philosophy*, *98*(2), 304–318.

15 Earp, B. D., Douglas, T., & Savulescu, J. (2017). Moral neuroenhancement. In S. Johnson & K. Rommelfanger (Eds.), *Routledge handbook of neuroethics* (pp. 166–184). Routledge.

16 Douglas, T. (2008). Moral enhancement. *Journal of Applied Philosophy*, *25*(3), 228–245.

17 Beck, B. (2015). Conceptual and practical problems of moral enhancement. *Bioethics*, *29*(4), 233–240.

18 Archer, A. (2016). Moral enhancement and those left behind. *Bioethics*, *30*(7), 500–510. See also Porsdam Mann, S., Earp, B. D., Nyholm, S., Danaher, J., Møller, N., Bowman‐Smart, H., Hatherley, J., Koplin, J., Plozza, M., Rodger, D., Treit, P. V., Renard, G., McMillan, J., & Savulescu, J. (2023). Generative AI entails a credit‐blame asymmetry. *Nature Machine Intelligence*, *5*(1), 472–475.

19 de Melo‐Martin, I., & Salles, A. (2015). Moral bioenhancement: Much ado about nothing? *Bioethics*, *29*(4), 223–232.

20 Kass, L. (2002). *Life, liberty and the defense of dignity: The challenge for bioethics*. Encounter Books; Sandel, M. J. (2007). *The case against perfection: Ethics in the age of genetic engineering*. Harvard University Press; Agar, N. (2010). *Humanity's end: Why we should reject radical enhancement*. MIT Press; Harris, J. (2020). Moral enhancement and freedom. In T. H. Murray & V. T. Chuan (Eds.), *The ethics of sports technologies and human enhancement* (pp. 399–408). Routledge.

21 Two quick clarifications. First, a charitable reconstruction of this template argument requires additional premises to get from C—the core idea—to a conclusion that recommends (ceteris paribus) against a given type of enhancement. Basically, even if one granted the soundness of the argument, they might still think that there are all‐things‐considered reasons to pursue or allow an enhancement (for example, on consequentialist grounds), *notwithstanding* the diminished value or praiseworthiness of the achievement at an individual level. See Box 1 for an application of this caveat to the debate on moral enhancement. Second, what it means for the value or praiseworthiness of something to be ‘undermined’ (as in C of the template argument) is ambiguous. It could mean that the thing loses *all* its value or praiseworthiness (i.e., the strongest version of the CAA) or it could mean that it loses *some* of its value or praiseworthiness, or a *considerable amount* and so on. We have used this ambiguous language, however, to reflect the ambiguity that is often present in various versions of the CAA as found in the bioethics literature. Even so, will try to disambiguate the relevant claims as needed for particular discussions.

22 Bradford, G. (2013). The value of achievements. *Pacific Philosophical Quarterly*, *94*(2), 204–224.

23 Greco, J. (2010). *Achieving knowledge: A virtue‐theoretic account of epistemic normativity*. Cambridge University Press, Ch. 6.

24 See, for example, Aristotle, *Nicomachean Ethics*, §1.7.

25 This line of thinking is closely related to *perfectionist* approaches in the philosophy of well‐being (e.g., Hurka 1996), though note that one could accept this type of rationale for P2 without signing on to the substantive commitments of perfectionism. For other discussions of the value of achievement understood as a kind of success through ability that is not shared by successes that are not connected in the right way with the exercise of ability, see, for example, Pritchard (2009) and Carter and Gordon (2014).

26 Kass, op. cit. note 20.

27 Sandel, op. cit. note 20.

28 Harris, op. cit. note 20.

29 Agar, op. cit. note 20.

30 It is worth noting that Agar focuses in particular on how our *emotional* appraisal of successes is impacted by enhancement use.

31 For a recent response to this type of argument in relation to moral enhancement, specifically, see Zahiu, A., Mihailov, E., Earp, B. D., Francis, K. B., and Savulescu, J. (2023) Empathy training through virtual reality: Moral enhancement with the freedom to fall? *Ethics and Information Technology*, *25*(50), 1–14.

32 For discussion on this point, see Schneider, A. J. (2018). William J. Morgan on fair play, treatment versus enhancement and the doping debates in sport. *Sport, Ethics and Philosophy*, *12*(4), 386–400; Gordon, E. C., & Dodds, C. (2023). High altitude, enhancement, and the ‘Spirit of Sport’. *Journal of the Philosophy of Sport*, *50*(1), 63–82.

33 For an overview discussion of moral enhancement, see, for example, Harris, J., & Savulescu, J. (2015). A debate about moral enhancement. *Cambridge Quarterly of Healthcare Ethics*, *24*(1), 8–22.

34 Note that while our discussion of P2‐ME suggests that the kind of value at issue in the argument is not merely subjectively perceived value, we take it that one could formulate an analogous version of the worry that would be consistent with meta‐normative commitments disallowing, for example, objective value.

35 As we were finalizing revisions on this paper, a preliminary empirical test of laypeople's attitudes toward something very like P2‐ME (the moral analogue of P2) was published in *Bioethics*: Lucas, S., Douglas, T., and Faber, N. S., (2024). How moral bioenhancement affects perceived praiseworthiness. *Bioethics*, *38*(2), 129–137. The authors found that ‘actions facilitated by morally bioenhanced individuals are considered *less deserving of praise* than similar actions facilitated by “traditional” moral enhancement—for example, moral self‐education. This diminished praise does not seem to be driven by an aversion to (moral) bioenhancement per se. Instead, it appears to be primarily attributable to a *perceived lack of effort* exerted by bioenhanced individuals in the course of their moral enhancement’ (p. 129). However, the examples used by the authors—for example, a surgically implanted ‘moral pacemaker’ or a specially programmed ‘subliminal’ moral enhancement TV set—are precisely the sorts of unrealistic, ‘direct’ moral enhancers that we object to in this paper. We are currently engaged in similar empirical research to assess lay attitudes towards realistic, “facilitative” enhancers such as the ones that we will be discussing herein.

36 It is worth pointing out a connection – but also some important differences—between the CAA as we conceive of it here and an authenticity‐based argument against moral enhancement as defended, for example, in Crutchfield, P. (2018). Moral enhancement can kill. *The Journal of Medicine and Philosophy*, *43*(5), 568–584. Crutchfield holds that ‘if moral enhancement manipulates a person's moral traits, and those moral traits constitute personal identity, then it is possible that moral enhancement could alter a person's identity’ (p. 568). If that argument is sound, it might seem to support the conclusion of the CAA, but by different argumentative means: basically, ‘you’ would not deserve praise for having achieved moral improvement (the value of it would not be creditable to you) since ‘you’ have been eliminated through the very moral enhancement that resulted in the alteration of your traits. But the CAA as we describe it is importantly distinct because, first, one might reasonably be persuaded by the cheapened achievement worry while remaining neutral on whether moral enhancement meaningfully changes the identity of the person whose moral traits are altered. Second, the two arguments also come apart in the opposite direction in that even if one is persuaded that differences in personal identity are implicated by moral trait manipulation (as per Crutchfield's argument), one might at the same time deny that, for example, achievement value interfaces with abilities and effort in the way that the achievement argument suggests.

37 Earp, B. D. (2018). Psychedelic moral enhancement. *Royal Institute of Philosophy Supplements*, *83*, 415–439.

38 Note that we do not take it that moral enhancements are distinct in that practical considerations could override (i.e., in favour of taking them) other competing considerations that might count against them. In this way, just as we suggest that a charitable construction of the *Template CAA (Moral Enhancement Version)* requires a ‘ceteris paribus’ qualification, the same would hold for the analogous argument in the case of cognitive or athletic enhancement. For discussion on this point, see Gordon, E. C. (2022). *Human enhancement and well‐being: A case for optimism*. Routledge.

39 Persson, I., & Savulescu, J. (2012). *Unfit for the future: The need for moral enhancement*. OUP Oxford.

40 Perhaps it could even be seen as a kind of moral *narcissism* to choose one's own more praiseworthy but slow and effortful moral enhancement over the more effective, quick intervention, which is easier.

41 Persson & Savulescu, op. cit. note 39.

42 Jotterand, F. (2011). ‘Virtue engineering’ and moral agency: Will post‐humans still need the virtues? *AJOB Neuroscience*, *2*(4), 3–9.

43 Sparrow, R. (2014). (Im)moral technology? Thought experiments and the future of ‘mind control’. In A. Akayabashi (Ed.), *The future of bioethics: International dialogues*. (pp. 113–119) Oxford University Press.

44 Ibid: 2, 3 (emphasis added).

45 Earp, B. D., et al., op. cit. note 15, p. 174.

46 Ibid.

47 Naar, H. (2016). Real‐world love drugs: Reply to nyholm. *Journal of Applied Philosophy*, *33*(2), 197–201.

48 Sparrow, op. cit. note 43.

49 Jotterand, op. cit. note 42.

50 From an anonymous reviewer—thank you. We have slightly edited the text for ease of interpretation.

51 Gilligan, J., & Lee, B. (2004). The psychopharmacologic treatment of violent youth. *Annals of the New York Academy of Sciences*, *1036*(1), 356–381.

52 Baccarini, E., & Malatesti, L. (2017). The moral bioenhancement of psychopaths. *Journal of Medical Ethics*, *43*(10), 697–701.

53 In general, psychedelics present a lower physiological risk profile than other psychoactive substances such as alcohol, with a very low potential for addiction. Adverse physical and psychological reactions such as feelings of nausea, anxiety or potentially more disturbing so‐called ‘bad trips’ do sometimes occur, although these appear to be mitigated by close attention to setting and preparation. Other risks relate to potential shifts in values, beliefs or sense of self that may be undesired and heightened vulnerability (e.g., to abuse) due to increased suggestibility. For discussions, see Schlag, A. K., Aday, J., Salam, I., Neill, J. C., & Nutt, D. J., (2022) Adverse effects of psychedelics: From anecdotes and misinformation to systematic science. *Journal of Psychopharmacology*, *36*(3), 258–272; McNamee, S., Devenot, N., & Buisson, M. (2023). Studying harms is key to improving psychedelic‐assisted therapy—Participants call for changes to research landscape. *JAMA Psychiatry*, *80*(5), 411–412; Romeo, B., Kervadec, E., Fauvel, B., Strika‐Bruneau, L., Amirouche, A., Verroust, V., Piolino, P., & Benyamina, A. (2024). Safety and risk assessment of psychedelic psychotherapy: A meta‐analysis and systematic review. *Psychiatry Research*, *335*, 115880.

54 Noorani, T., Garcia‐Romeu, A., Swift, T. C., Griffiths, R. R., & Johnson, M. W. (2018). Psychedelic therapy for smoking cessation: Qualitative analysis of participant accounts. *Journal of Psychopharmacology*, *32*(7), 756–769.

55 Ibid: 759.

56 Ibid: 760.

57 As reviewed and discussed in, e.g., Ahlskog, R. (2017). Moral enhancement should target self‐interest and cognitive capacity. *Neuroethics*, *10*, 363–373; Earp, op. cit. note 37; Gordon, op. cit. note 11; Lange, V., & Marie, S. (2021). Exploring moral bio‐enhancement through psilocybin‐facilitated prosocial effects. *Journal of Cognition & Neuroethics*, *8*(1); Kirkham, N., & Letheby, C. (2024). Psychedelics and environmental virtues. *Philosophical Psychology*, *37*(2), 371–395; Rakić, V. (2023). Psilocybin: The most effective moral bio‐enhancer? *Bioethics*, *37*(7), 683–689; Kähönen, J. (2023). Psychedelic unselfing: Self‐transcendence and change of values in psychedelic experiences. *Frontiers in Psychology*, *14*, 1104627.

58 Griffiths, R. R., Richards, W. A., McCann, U., & Jesse, R. (2006). Psilocybin can occasion mystical‐type experiences having substantial and sustained personal meaning and spiritual significance. *Psychopharmacology*, *187*, 268–283.

59 Persson & Savulescu, op. cit. note 39.

60 Ahlskog, op. cit. note 57.

61 Kähönen, op. cit. note 57, abstract.

62 Kirkham & Letheby, op. cit. note 57. See also Lyons, T., & Carhart‐Harris, R. L. (2018). Increased nature relatedness and decreased authoritarian political views after psilocybin for treatment‐resistant depression. *Journal of psychopharmacology*, *32*(7), 811–819; Sagioglou, C., & Forstmann, M. (2022). Psychedelic use predicts objective knowledge about climate change via increases in nature relatedness. *Drug Science, Policy and Law*, *8*, 20503245221129803; Li, J. (2023). The mediating role of personality traits in psychedelics' relationships with nature connectedness and pro‐environmentalism. *NHSJS Reports*, 1–11,

63 For recent discussions of the ethical implications of using hallucinogenic versus non‐hallucinogenic psychedelics as the standard of care in clinical contexts, see Cheung, K., Patch, K., Earp, B. D., & Yaden, D. B. (2023). Psychedelics, meaningfulness, and the “Proper Scope” of medicine: Continuing the conversation. *Cambridge Quarterly of Healthcare Ethics*, 1–7; Yaden, D. B., Earp, B. D., & Griffiths, R. R. (2022). Ethical issues regarding nonsubjective psychedelics as standard of care. *Cambridge Quarterly of Healthcare Ethics*, *31*(4), 464–471. See also Rasmussen, K., & Olson, D. E. (2022). Psychedelics as standard of care? Many questions remain. *Cambridge Quarterly of Healthcare Ethics*, *31*(4), 477–481; Peterson, A., & Sisti, D. (2022). Skip the trip? Five arguments on the use of nonhallucinogenic psychedelics in psychiatry. *Cambridge Quarterly of Healthcare Ethics*, *31*(4), 472–476.

64 Noorani, T., et al., op. cit. note 54, p. 759.

65 Yaden, D. B., Potash, J. B., & Griffiths, R. R. (2022). Preparing for the bursting of the psychedelic hype bubble. *JAMA Psychiatry*, *79*(10), 943–944.

66 Pace, B. A., & Devenot, N. (2021). Right‐wing psychedelia: Case studies in cultural plasticity and political pluripotency. *Frontiers in Psychology*, *12*, 733185.

67 Zahiu, A., et al. op. cit. note 31.

68 Ibid: 50.

69 Dunivan, D. W., Mann, P., Collins, D., & Wittmer, D. P. (2024). Expanding the empirical study of virtual reality beyond empathy to compassion, moral reasoning, and moral foundations. *Frontiers in Psychology*, *15*, 1402754, abstract, emphasis added.

70 Graham, J., Nosek, B. A., Haidt, J., Iyer, R., Koleva, S., & Ditto, P. H. (2011). Mapping the moral domain. *Journal of Personality and Social Psychology*, *101*(2), 366–385.

71 Lara, F., & Deckers, J. (2020). Artificial intelligence as a socratic assistant for moral enhancement. *Neuroethics*, *13*(3), 275–287.

72 Giubilini, A., Porsdam Mann, S., Voinea, C., Earp, B. D., & Savulescu, J. (2024). Know thyself, improve thyself: Personalized llms for self‐knowledge and moral enhancement. Pre‐print. https://papers.ssrn.com/sol3/papers.cfm?abstract_id=4863758

73 Savulescu, J., & Maslen, H. (2015). Moral enhancement and artificial intelligence: Moral AI? In J. Romportl, E. Zackova, & J. Kelemen (Eds.), *Beyond artificial intelligence: The disappearing human‐machine divide* (pp. 79–95). Springer; Klincewicz, M. (2016). Artificial intelligence as a means to moral enhancement. *Studies in Logic, Grammar and Rhetoric*, *48*(1), 171–187; Volkman, R., & Gabriels, K. (2023). AI moral enhancement: Upgrading the socio‐technical system of moral engagement. *Science and Engineering Ethics*, *29*(2), 11; Demaree‐Cotton, J., Earp, B. D., & Savulescu, J. (2022). How to use AI ethically for ethical decision‐making. *The American Journal of Bioethics*, *22*(7), 1–3; Giubilini, A., & Savulescu, J. (2018). The artificial moral advisor. The “Ideal Observer” meets artificial intelligence. *Philosophy & technology*, *31*, 169–188.

74 Savulescu & Maslen, op. cit. note 73.

75 Lara & Deckers, op. cit. note 71.

76 But see Smith, W. R., & Appelbaum, P. S. (2021). Two models of legalization of psychedelic substances: Reasons for concern. *JAMA*, *326*(8), 697–698.

77 Note that the falsity of PI‐ME** is compatible with the idea that the level of effort on display across different cases of adjunctive–facilitative moral enhancement can vary. PI‐ME** is true only if such enhancement always “undermines” the moral success being attained through “substantial” ability and effort. Our cases show that there will be plenty of occasions, and indeed in paradigmatic cases of adjunctive–facilitative enhancement, where the ability and effort on display likely will be substantial enough (or again, even amplified) that it would be strange to conclude that they had been “undermined” in any relevant sense.

78 Maslen, H., et al., op. cit. note 14.

